# Automated Identification of Coronary Arteries in Assisting Inexperienced Readers: Comparison between Two Commercial Vendors

**DOI:** 10.3390/diagnostics12081987

**Published:** 2022-08-16

**Authors:** Domenico De Santis, Giuseppe Tremamunno, Carlotta Rucci, Tiziano Polidori, Marta Zerunian, Giulia Piccinni, Luca Pugliese, Benedetta Masci, Nicolò Ubaldi, Andrea Laghi, Damiano Caruso

**Affiliations:** Department of Medical Surgical Sciences and Translational Medicine—Sapienza, University of Rome, Radiology Unit—Sant’Andrea University Hospital, Via di Grottarossa 1035-1039, 00189 Rome, Italy

**Keywords:** CCTA, automated coronary analysis, coronary arteries, coronary artery disease

## Abstract

Background: to assess the performance and speed of two commercially available advanced cardiac software packages in the automated identification of coronary vessels as an aiding tool for inexperienced readers. Methods: Hundred and sixty patients undergoing coronary CT angiography (CCTA) were prospectively enrolled from February until September 2021 and randomized in two groups, each one composed by 80 patients. Patients in group 1 were scanned on Revolution EVO CT Scanner (GE Healthcare), while patients in group 2 had the CCTA performed on Brilliance iCT (Philips Healthcare); each examination was evaluated on the respective vendor proprietary advanced cardiac software (software 1 and 2, respectively). Two inexperienced readers in cardiac imaging verified the software performance in the automated identification of the three major coronary vessels: (RCA, LCx, and LAD) and in the number of identified coronary segments. Time of analysis was also recorded. Results: software 1 correctly and automatically nominated 202/240 (84.2%) of the three main coronary vessels, while software 2 correctly identified 191/240 (79.6%) (*p* = 0.191). Software 1 achieved greater performances in recognizing the LCx (81.2% versus 67.5%; *p* = 0.048), while no differences have been reported in detecting the RCA (*p* = 0.679), and the LAD (*p* = 0.618). On a per-segment analysis, software 1 outperformed software 2, automatically detecting 942/1062 (88.7%) coronary segments, while software 2 detected 797/1078 (73.9%) (*p* < 0.001). Average reconstruction and detection time was of 13.8 s for software 1 and 21.9 s for software 2 (*p* < 0.001). Conclusions: automated cardiac software packages are a reliable and time-saving tool for inexperienced reader. Software 1 outperforms software 2 and might therefore better assist inexperienced CCTA readers in automated identification of the three main vessels and coronaries segments, with a consistent time saving of the reading session.

## 1. Introduction

Coronary artery disease (CAD) is the most frequent heart disease and is considered the leading cause of death in US citizens, accounting for more than 600 thousand deaths yearly [1]. The underlying etiology of CAD includes atherosclerosis, which consists of endothelial disruption and subsequent lipid accumulation and deposition in the intima layer, causing luminal narrowing [2]. Coronary computed tomography angiography (CCTA) is a non-invasive imaging modality recommended as the first diagnostic test for CAD in symptomatic patients and in patients with low to intermediate risk of obstructive CAD that cannot be excluded by clinical assessment alone, according to European Society of Cardiology (ESC) 2019 guidelines [3]. The importance of CCTA lies in its ability to rule out coronary stenosis with a negative predictive value (NPV) ranging between 97% and 99%, whilst ensuring the visualization of the vessel wall and plaque morphology [4,5,6].

The assessment of coronary arteries patency involves vessel extraction and visual assessment, which can be rather subjective, expert-dependent, and time consuming, due to image noise, vessel bifurcation or irregular pathway, and uneven image signal [7,8]. Furthermore, for a correct evaluation of the coronary arteries, orthogonal cross sections, curved multiplanar reformation (MPR), and volume rendering techniques (VRT) reconstructions are required. Manual vessel identification and segmentation are time-consuming and require expertise; automatic tools are under ongoing development [9], providing benefits in terms of speed and accuracy in vessel detection [10].

Although correct interpretation of CCTA requires adequate training and experience [11,12], most practitioners have little exposure to CCTA interpretation during their medical training years [13]; therefore, the implementation of automated steps in the reading sessions appears to be desirable. Automated coronary analysis software packages provide vessel segmentation without human intervention, allowing time-effective fully automated identification of the coronary tree [14]. Compared to manual segmentation, these software significantly reduce reconstruction time, including those cases in which brief manual input is necessary due to incomplete automated coronary vessels segmentation [9]. The application of such tools are more convenient than semi-automated methods and may theoretically further facilitate coronary vessels’ evaluation, favoring the trend towards less reader dependency [15]. It has been demonstrated that advanced cardiac software packages have a NPV similar to high experience readers while their positive predictive value is comparable to novice CCTA readers [16]; additionally, they may assist novice readers in ruling out significant CAD [17]. Nevertheless, to the best of our knowledge, previous investigations were focused on diagnostic performances rather than the identification of coronary vessels and segments as an aiding tool for inexperienced CCTA readers.

Therefore, in this prospective study, we evaluated and compared the performance and speed of two commercially available advanced cardiac software packages in the automated identification of coronary vessels as an aiding tool for inexperienced readers.

## 2. Materials and Methods

### 2.1. Patient Population

This prospective study was approved by local institutional review board and written informed consent was obtained from all patients. From February 2021 until September 2021 consecutive patients undergoing elective CCTA for known or suspected CAD were enrolled. Patients with contraindication to iodine contrast medium injection, renal insufficiency (eGFR < 30 mL/min/1.73 m^2^), previous bypass grafts or coronary stenting, and unstable angina pectoris, were not included in this study. Patients with a heart rate (HR) > 75 bpm were treated with intravenous beta-blocker (metoprolol tartrate, 5 mg). Nitrates were sublingually administrated to all patients, after exclusion of contraindications, to induce vasodilation (Trinitrine, 0.8 mg).

Patients were randomly assigned (1:1 ratio, blocks of ten) into two groups: patients in group 1 had the CCTA acquired on GE Revolution EVO CT Scanner (GE Healthcare, Milwaukee, WI, USA), while patients in group 2 had the CCTA performed on Brilliance iCT (Philips Healthcare, Cleveland, OH, USA). Each examination was eventually evaluated on the respective vendor proprietary advanced cardiac software.

### 2.2. Image Acquisition—Group 1

CCTA examinations of group 1 were performed with a 128-slice CT (GE Revolution EVO CT Scanner, GE Healthcare, Milwaukee, WI, USA) in cranio-caudal direction. CT images were acquired using a retrospective ECG-gated protocol with the following scan parameters: tube voltage of 120 kVp (in patients with BMI ≥ 30 kg/m^2^) or 100 kVp (in patients with BMI < 30 kg/m^2^), tube current of 200 mAs, detector collimation of 0.625 mm, gantry rotation time of 0.35 s, adaptive pitch factor varying from 0.16 to 0.23 based on heart rate, and matrix size of 512 × 512 pixel.

All patients received 70 mL of intravenous of non-ionic high-iodine concentration contrast medium (400 mg I/mL iomeprol, Iomeron 400; Bracco Imaging, Milan, Italy) at flow rate of 5 mL/s through an 18-gauge antecubital access, by using an automated triple-syringe power injector (MEDRAD^®^ Centargo CT Injection System; Bayer AG, Berlin, Germany), followed by saline chaser bolus of 30 mL at the same flow rate. Scan delay was determined using a bolus-tracking software program (SmartPrep, GE Healthcare, Milwaukee, WI, USA), images were acquired 6 **s** after the trigger attenuation threshold (150 HU) was reached into a region-of-interest (ROI) placed in the ascendent aorta at the level of pulmonary arteries.

The CCTA of each patient was reconstructed at 75% phase of the R-R interval of the cardiac cycle with hybrid IR reconstruction (ASiR-V, GE Healthcare) at a strength level of 50%, using standard kernel. The following specifications were applied: matrix size of 512 × 512 pixels; reconstruction field of view of 250 mm; section thickness of 0.625 mm; and increment of 0.625 mm.

### 2.3. Image Post Processing—Group 1

**Figure 1:**
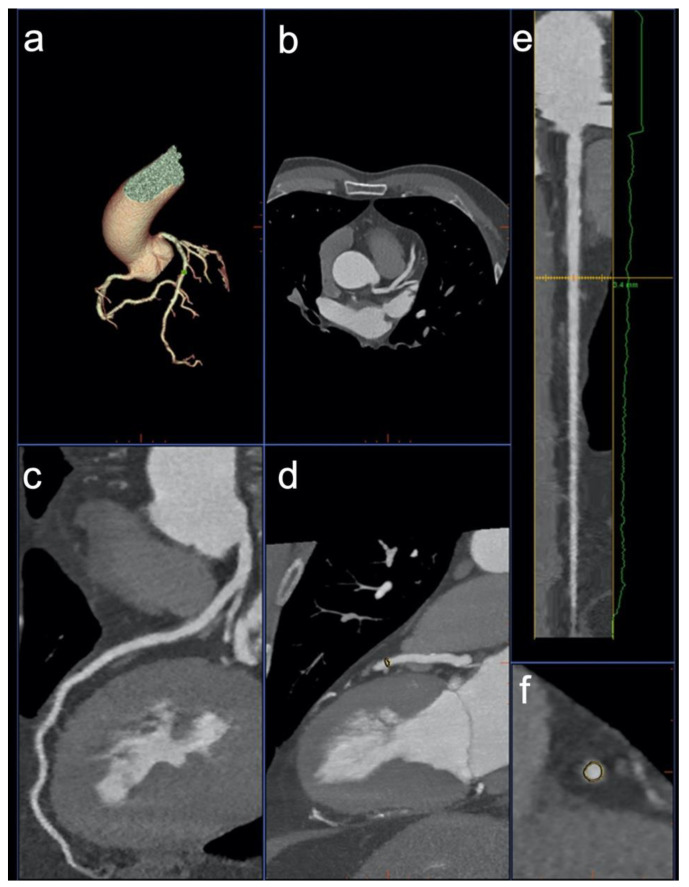
Software 1 showing a VR of the coronary artery tree (**a**), axial images (**b**), curved multiplanar reformation (cMPR, **c**), and maximum intensity projection (**d**) of the LAD. Additionally, the software generates a stretched cMPR along the centerline (**e**) and axial sections of the vessel lumen (**f**).

CCTA images were transferred to a three-dimensional multimodality workstation (Advantage Workstation 4.7, GE Healthcare) for further analysis. After dataset selection, the application “CardIQ Xpress” was launched, selecting the “auto coronary analysis” tool. The application is designed to automatically label the coronary vessels and to present axial images, curved MPR, VRT and lumen views of all the identified arteries (Figure 1).

### 2.4. Image Acquisition—Group 2

CCTA examinations of group 2 were performed with a 256-slice CT (Brilliance iCT; Philips Healthcare, Cleveland, OH, USA) in cranio-caudal direction. CT images were acquired using a retrospective ECG-gated protocol with the following scan parameters: tube voltage of 120 kVp (in patients with BMI ≥ 30 kg/m^2^) or 100 kVp (in patients with BMI < 30 kg/m^2^), tube current of 800 mAs, detector collimation of 128 × 0.625 mm, gantry rotation time of 0.27 s, pitch of 0.16, and matrix size of 512 × 512 pixels. Contrast media injection protocol was identical to what previously described in group 1. Scan delay was determined using a bolus-tracking software program (Bolus Tracking, Philips Healthcare), images were acquired 6 **s** after the trigger attenuation threshold (150 HU) was reached into a region-of-interest (ROI) placed in the ascendent aorta at the level of pulmonary arteries.

The CCTA of each patient was reconstructed at 75% phase of the R-R interval of the cardiac cycle with IR reconstruction (iDose^4^, Philips Healthcare) at a strength level 7, using Xres standard (XCB) kernel. The following specifications were applied: matrix size of 512 × 512 pixels; reconstruction field of view of 220 mm; section thickness of 0.8 mm; and increment of 0.4 mm.

### 2.5. Image Post Processing—Group 2

**Figure 2:**
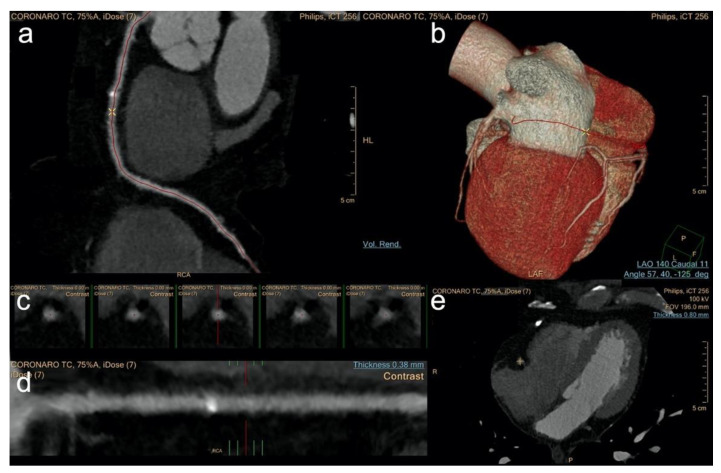
Software 2 showing cMPR of the RCA (**a**), a VR model of the whole heart (**b**), axial sections of the vessel lumen (**c**), a stretched cMPR along the centerline (**d**), and axial image of the middle segment of vessel (**e**).

CCTA images were transferred to a three-dimensional multimodality workstation (IntelliSpace Portal v6.0.2.33500, Philips Healthcare) for further analysis. After dataset selection, the application “Comp. Cardiac” was launched. The application is designed to automatically label the coronary vessels, the aorta, heart chambers and left ventricle myocardium. For the purpose of our study, only the coronary arteries were selected and displayed. The software subsequently displays axial images, curved MPR, VRT, and lumen views of all the identified arteries (Figure 2).

### 2.6. Image Analysis

Automated detection of the coronary arteries was performed using the aforementioned one-click coronary detection tools available at the two workstations. Two inexperienced readers in cardiac imaging (1-year radiology residents) who had previously received training in operating both software by a board-certified radiologist with ten years of experience in CCTA, assessed software performance in the automated identification of the three major coronary vessels [right coronary artery (RCA), left circumflex (LCx), and left anterior descending (LAD)] and in the number of identified coronary segments according to a 16-segments modified version [6] of the Society of Cardiovascular Computed Tomography (SCCT) model [18]: segments 1–14, coronary artery segments of the RCA, LAD and LCx as initially described; segment 15, combined left posterior descending and posterolateral branches; segment 16, ramus intermedius. Time of analysis was also recorded for both software. Results were eventually validated by a board-certified radiologist with 5 years of experience in cardiovascular imaging.

### 2.7. Statistical Analysis

Statistical comparisons were performed using MediCalc^®^ Statistical Software version 20.014 (MedCalc Software Ltd., Ostend, Belgium; https://www.medcalc.org; 2021).

Variables were expressed as mean ± standard deviation or as frequency and percentage, as appropriate. Differences between patient characteristics in group 1 and 2 and differences in software processing time were assessed by Student’s t-test. Differences between software performances in the identification of coronary vessels and segments in group 1 and 2 were assessed with the “N-1” Chi-squared test [19,20]. A *p* value < 0.05 was considered to indicate a statistically significant result.

## 3. Results

### 3.1. Patient Population

**Figure 3:**
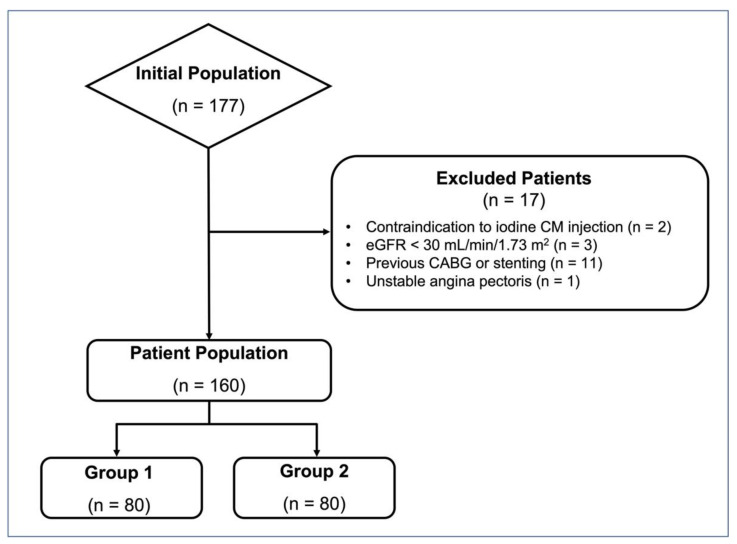
Flow diagram of patient recruitment (CM: contrast medium; eGRF: estimated glomerular filtration rate; CABG: coronary artery bypass grafting).

Detailed results of patient characteristics are reported in Table 1, flow diagram of patient recruitment is depicted in Figure 3. Final study population included 160 patients: 80 patients scanned on GE Revolution EVO CT Scanner (group 1) and 80 patients scanned on Philips Brilliance iCT (group 2). Mean age was 63 ± 11 years in group 1 and 64 ± 12 years in group 2 (*p* = 0.583). Most of the population (96/160; 60%) was composed by male, with a higher proportion in both groups: 50 individuals (63%) in group 1 and 46 individuals (58%) in group 2 (*p* = 0.519). Mean BMI was 28.3 ± 5.5 kg/m^2^ in group 1 and 27.7 ± 4.4 kg/m^2^ in group 2 (*p* = 0.675). Mean HR measured during the examination was 58 ± 7 bpm in group 1 and 59 ± 7 bpm in group 2 (*p* = 0.367).

### 3.2. Image Analysis

**Figure 4:**
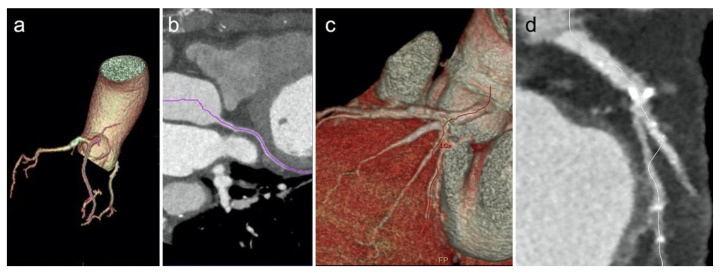
Software 1 showing a VR model (**a**) and a cMPR (**b**) of a correctly identified LCx. Software 2 (**c**,**d**) provides an inaccurate vessel identification, tracking part of the first obtuse marginal artery in lieu of the proximal LCx.

Comprehensive results on software performances are reported in Table 2. On a per-vessel analysis, software 1 and software 2 did not show statistically significant differences in their performances, correctly identifying a comparable number of coronary vessels (*p* = 0.191). In particular, software 1 achieved greater performances than software 2 in recognizing the LCx (*p* = 0.048; Figure 4), while no differences have been reported in detecting the RCA (*p* = 0.679) and the LAD (*p* = 0.618).

On a per-segment analysis, software 1 outperformed software 2, automatically detecting 88.7% of the coronary segments, while software 2 detected 73.9% (*p* < 0.001).

Average reconstruction and detection time was 13.8 s for software 1 and 21.9 s for software 2 (*p* < 0.001).

## 4. Discussion

The aim of our investigation was to assess the performances two vendor proprietary software in the identification of coronary vessels, as aiding tool for inexperienced CCTA readers. Our results demonstrated both software had similar performances in terms of number of identified vessels, while software 1 identified 14.8% more coronary segments (*p* < 0.001) and was 37% faster than software 2 (*p* < 0.001).

Correct interpretation of cardiac imaging requires extensive training and experience, needed to reduce inter-observer variability [21]. Inexperienced readers, at the beginning of their learning curve, benefit from the assistance of dedicated software in the identification of coronary arteries and segments [17,22,23]. A thorough evaluation of the coronary tree is fundamental for the CCTA to achieve high diagnostic yield. Native axial CCTA images can be rapidly assessed during the reporting session; however, an accurate evaluation of the coronary tree cannot simply rely on standard axial image datasets. MPRs are fundamental to validate stenoses on a second plane and, therefore, to avoid false positive findings. Curved MPRs and true axial lumen views are of fundamental importance to comprehensively evaluate the presence CAD, by depicting coronary stenoses in their full extent [24].

Software reliability in correctly identifying and labeling the coronary arteries is mandatory in the diagnostic setting, especially for readers with little experience, often in-training and not extensively exposed to CCTA interpretation sessions. Our results demonstrated the LAD was the coronary vessel most frequently identified in both groups (90% and 87.5% in group 1 and 2, respectively), such performance might be explained by the fact that the LAD is the coronary vessel least affected by overall motion during the cardiac cycle [25]. Both software packages returned also comparable performance in the identification of the RCA (*p* = 0.679), while software 1 performed significantly better in the identification of the LCx (*p* = 0.048); a possible explanation of this result might be related to LCx smaller caliber compared to other major vessels in the high prevalent setting of right-dominant circulation [26]; therefore, software 2 performance might be negatively influenced by vessel diameter compared to software 1. Despite these small differences, both software packages had similar performances in the overall identification of the coronary tree (*p* = 0.191). Nevertheless, focusing on the per-segment analysis, software 1 outperformed software 2, identifying 14.8% more coronary segments (*p* < 0.001); these results demonstrate that both software packages might assist inexperienced readers during the CCTA reporting session.

In terms of time-effectiveness, software 1 was 37% faster that software 2 (*p* < 0.001). Although time of analysis is not a primary endpoint when reporting a CCTA, time plays an important role both in clinical workflow and in determining procedural reimbursement [21]; therefore, a fast and reliable coronary vessel identification is highly advisable in clinical practice, especially for readers at the beginning of their learning curve, typically slower and less accurate than more experienced readers in the interpretation of CCTA datasets [11,21,27].

Further, the field artificial intelligence (AI) has recently gained momentum in radiology. In particular, machine learning (a subset of AI) and deep learning (a subset of machine learning) are currently under active investigation both in private sectors and in several academic institutions worldwide, showing potential for clinical workflow optimization, image and reporting improvements. The implementation of AI in cardiac imaging is also achieving promising results in prognosis prediction, automated calcium score quantification, plaque characterization and stenosis evaluation [28,29,30,31,32,33]. In this regard, AI can also be applied to coronary artery segmentation in CCTA, further assisting inexperienced readers in their diagnostic tasks. However, vessel segmentation is only the first step of a comprehensive CCTA evaluation; therefore, additional investigations are needed obtain a seamless integration of AI in clinical practice [34].

Our study has some limitations that should be addressed. First, it was limited to the two vendors that are available at our Institution; other software packages might outperform the ones we have investigated; therefore, comparisons with other vendors are advisable. Second, our investigation was not designed on diagnostic accuracy; nevertheless, automated approaches in stenosis detection have been proved effective in improving inexperienced readers’ sensitivity in diagnostic CAD [35]. Third, in our study population no patient had coronary artery origin anomalies, which might represent a challenge for the software in the correct vessel identification.

## 5. Conclusions

In conclusion, our results suggest that automated cardiac software packages are a reliable and time-saving tool for inexperienced readers. Software 1 outperforms software 2 and might, therefore, better assist inexperienced CCTA readers in automated identification of the three main vessels and coronary segments, with a consistent time saving of the reading session. Since correct vessel identification is also a fundamental step of CCTA interpretation; these software packages might also improve inexperienced readers’ diagnostic performances through the identification of coronary stenoses.

## Figures and Tables

**Table 1:** Patient Characteristics.

	Group 1	Group 2	*p* Value
Patient Characteristics			
Age, y *	63 ± 11	64 ± 12	0.583
BMI *	28.3 ± 5.5	27.7 ± 4.4	0.675
HR *	58 ± 7	59 ± 7	0.367
Sex: Male ^†^	50 (63)	46 (58)	0.519
Sex: Female ^†^	30 (38)	34 (43)	0.520
Cardiovascular Risk Factors ^†^			
Family history of CAD	57 (71.3)	56 (70)	0.857
Hypertension	53 (66.3)	56 (70)	0.616
Hypercholesterolemia	33 (41.3)	40 (50)	0.270
Diabetes Mellitus	24 (30)	16(20)	0.145
Current of former smoking	29 (36.3)	45 (56.3)	0.011
Medications ^†^			
Beta-blockers	24 (30)	19 (23.8)	0.378
Nitrates	33 (41.3)	46 (57.5)	0.004

* Data are mean ± SD. ^†^ Data are number of patients (%).

**Table 2:** Software performances.

	Group 1	Group 2	*p* Value
RCA	65/80 (81.2)	67/80 (83.7)	0.679
LAD	72/80 (90)	70/80 (87.5)	0.618
LCx	65/80 (81.2)	54/80 (67.5)	0.048
RCA–LCx–LAD	202/240 (84.2)	191/240 (79.6)	0.191
Coronary Segments	942/1062 (88.7)	797/1078 (73.9)	<0.001
Time of analysis	13.8 ± 2 s	21.9 ± 3 s	<0.001

LAD: left anterior descending artery; LCx: left circumflex artery; RCA: right coronary artery.

## Data Availability

Not applicable.
